# History and development of medical studies at the University of Witten/Herdecke – an example of “continuous reform”

**DOI:** 10.3205/zma001269

**Published:** 2019-10-15

**Authors:** Katja Frost, Friedrich Edelhäuser, Marzellus Hofmann, Diethard Tauschel, Gabriele Lutz

**Affiliations:** 1 University of Witten/Herdecke, Faculty for Health, Department of Human Medicine, Witten, Germany

**Keywords:** model study course, student centered education, students as co-designers of medical studies, „Masterplan 2020“, practice oriented medical education

## Abstract

**Introduction: **The University of Witten/Herdecke (UW/H) was founded in 1982 as the first privately run German university. In addition to economics, dentistry, a center for life sciences and the institute for general studies, the main focus from the inception of the University was the development of a model course in medical studies.

**Methodology: **A description of the history of the development of medical studies in relation to the reasons for its founding, its founding ideals and their implementation; phases of development, transformations and influencing factors are presented in detail. External assessments are also used for this purpose.

**Result: **The “Herdecke Model” was first implemented with the initial group of medical students in 1983. In the past 36 years the curriculum for medical education in Witten/Herdecke has evolved to meet internal and external requirements. The goals of the founders for a reform of medical studies and the founding ideals of the UW/H have continued to lead the University through a continuous reform process of medical training. From the first model of a reform degree course at UW/H 1983 to the current Model Study Course/Modellstudiengang (MSG 2018^plus^), these reforms have manifested themselves in four major phases spanning a 15 year period.

Landmarks of the reforms include the first systematic introduction of problem-oriented learning in Germany, and clinical and practical training with real patients in both clinical and general medical elective blocks that far surpass the Medical Licensure Act’s requirements. Additionally noteworthy are the introduction of PY training wards and the active participation and co-design role students may hold.

**Discussion: ** Due to the small size of UW/H, reforms can be tested quickly and implemented with ease and flexibility. This facilitates a “laboratory setting” for the testing of future-oriented innovations. The small size has allowed various concepts to be able to be used as models, thus serving as stimuli for larger change in medical studies in Germany.

## Zusammenfassung

**Einleitung: **Die Universität Witten/Herdecke wurde 1982 als erste deutsche Universität in freier, privater Trägerschaft gegründet. Neben den Wirtschaftswissenschaften, der Zahnmedizin, einem Zentrum für Biowissenschaften und dem Institut für das Studium fundamentale galt ein Hauptaugenmerk von Anfang an der Entwicklung eines modellhaften Medizinstudiums.

**Methodik: **Die Entwicklungsgeschichte des Medizinstudiums wird ausgehend von den Gründungsimpulsen und –idealen und deren Implementierung deskriptiv verfolgt. Phasen der Entwicklung, Transformationen und einflussnehmende Faktoren werden herausgearbeitet. Dazu werden auch externe Beurteilungen herangezogen.

**Ergebnis: **Bereits mit dem Start des ersten Jahrgangs Medizin 1983 wurde das „Modell Herdecke“ erstmals umgesetzt. In den seither vergangenen 36 Jahren hat sich das Curriculum der ärztlichen Ausbildung in Witten/Herdecke auf innere und äußere Anforderungen hin weiterentwickelt.

Die Anliegen der Gründer für eine Reform des Medizinstudiums und die Gründungsideale der Universität Witten/Herdecke wirkten durch einen kontinuierlichen Reformprozess der ärztlichen Ausbildung in Witten/Herdecke fort und manifestierten sich in insgesamt vier größeren Reformabschnitten vom ersten Modell- bzw. Reformstudiengang in Witten 1983 bis zum aktuellen Modellstudiengang MSG 2018^ plus^.

Zu den Besonderheiten gehören insbesondere die deutschlandweit erste systematische Einführung des Problem-orientierten Lernens, eine weit über die Anforderungen der ÄAppO hinausgehende klinische und praktische Ausbildung mit realen Patienten in klinischen und allgemeinmedizinischen Blockpraktika sowie die Einführung von PJ-Ausbildungsstationen. Ein Kernfaktor scheint auch die aktive Partizipation und Mitgestaltung von Studierenden zu sein.

**Diskussion: **Durch die geringe Größe können Reformideen in Witten/Herdecke schnell erprobt und flexibel umgesetzt werden. Dies ermöglichte einen „Laborstatus“ für die Erprobung von zukunftsfähigen Neuerungen. Trotz der „Kleinheit“ des Medizinstudiums in Witten/Herdecke wurde dabei auf einen „Modellcharakter“ der Konzeptionen geachtet und es gingen von dort Impulse für die Veränderung des Medizinstudiums in Deutschland aus.

## 1. Introduction

### 1.1. Foundation of the university and planning of reform-oriented medical studies 

The concept for the foundation of Witten/Herdecke University (UW/H) as a newly conceived private university that is largely independent of state regulations goes back a long way. It emerged in 1945/46 from an anthroposophical student group in Tübingen, which formed immediately after the catastrophe of the Second World War. Gerhard Kienle was the founding spiritual leader of the group, retaining this role until his death in June 1983. In Kienle’s view both the experience of the barbarity of war, and the knowledge of the cruel medical practices that treated people solely as scientific objects demanded a clear counter-model. The central concern was the “development of culturally effective institutions that can facilitate free intellectual life” [1]. With regards to the healthcare system this would allow focusing on the dignity and individuality of the sick person. 

 A milestone along the journey to implement reform-oriented medical education was the opening of the “Gemeinschaftskrankenhaus Herdecke” in November 1969. The Hospital implemented a structural emphasis on interprofessional cooperation on the basis of patient-centered anthroposophical medicine [2]. This impact was accompanied by fostering international academic cooperation: in 1973 a symposium entitled “Toward a Man Centered Physiological Science and Medicine” took place in Herdecke with the participation of internationally renowned scientists [[3] and [4], p. 506]. In 1976 the Free European Academy of Sciences was founded [[4], p. 509]. The development of a new type of medical studies was birthed from this foundation. This same year – still without state approval – a preparatory medical course began which initiators (even in 1976) dubbed a “model study course” [[4], p. 509]. Political resistance and formal legal reasons prevented the University from opening during these years. However the participants pursued their common goal with great commitment, and were able to successfully convince important decision makers in politics and business in the years following. 

Shortly after the opening of the University but before the first cohort of medical students could begin their studies, Gerhard Kienle fell ill, and died soon afterwards. Konrad Schily was appointed President of the University from 1983-1999 and 2002-2004, thus became instrumental in the implementation of the University project [5]. 

## 2. Implementation of the reform concept

Since the opening of the University 36 years ago, its guiding principles have encouraged the involvement of all stakeholders and interest groups, the students in particular. The appreciation of differing perspectives – at times through vigorous discussion – has allowed for a continuous and widely supported reform process. 

### 2.1. “Freedom, truth and responsibility” as the foundation of continuous development

At the Dean’s Symposium in Munich on 18 October 1982, Gerhard Kienle stated the following regarding the founding reasons and methodically guiding ideals for the continuous development of scientific studies at Witten/Herdecke University of (UW/H): “Truth, freedom and social responsibility in their full unconditionality are the Herdecke model.” [[6], p. 354]:

- The pursuit of truth is understood as an expression of an “unconditional love for the cause” [[6], pp. 70-71].
- The guarantee of personal freedom is named as a condition and prerequisite for scientific work in research, teaching and university studies.
- Ultimately, social responsibility consists of aligning one’s pursuit of knowledge in such a way that it will benefit others, and strengthen and promote the value and dignity of fellow human beings in society.

These values remain central to the University's curriculum. The triad of the basic curriculum tenets are knowledge, skills and values. It is values and attitudes that are given the most importance. The centrality of these basic values highlights how seriously they are honoured. 

#### 2.2. Controversy as the engine of a beneficial development

Soon after UW/H was founded in 1985, the first signs of conflict emerged around the introduction of modern teaching and learning methods. This tension arose between the more interdisciplinary vision of the Deanery and the more subject-oriented vision of the departmental representatives. Robert Wiedersheim, the then Dean of the Medical Faculty and former WHO department head for Health Manpower Development [7], moderated the development and change process. Wiedersheim deemed this process necessary and involved not only the departmental representatives, but above all the students. Wiedersheim considered the controversy implied in the process as essential to aid the development of a modern curriculum that was broadly accepted across the entire faculty. He introduced the curriculum designers of the University to an international network of reform universities: McMaster (Canada), Maastricht (The Netherlands), and Linköping (Sweden). Furthermore, he introduced them to international forums on questions regarding medical education (AMEE, GMA) and sought contact with the “Murrhardt circle” [8], [9].

The inclusion and appreciation of differing interests and perspectives has remained a key factor of the University's development and change processes in ensuing years. 

#### 2.3. Students as co-designers and co-responsible people

The “Witten Didactics” had repeated and significant design input from the students who also supported and further developed the implementation. Students in Witten were and are encouraged and empowered to take responsibility for the continuing development of the university.

The general willingness to co-design and assumption of responsibility is – in addition to motivation for medical studies, suitability and ability to reflect – one of the criteria in the UW/H selection process. This conferred responsibility is exemplified by the involvement of experienced students in the selection process, in teaching, in curriculum development and in committee work at all levels. Through the StudierendenGesellschaft (a non-profit organization), students form part of the University’s shareholders and manage the entire tuition fee financing arrangement. This allows for a free medical degree course while studying by means of a reverse generational contract. The StudierendenGesellschaft is the direct “mouthpiece of the students in the highest commercial body of the University – a first in Germany.” [https://studierendengesellschaft.de/], [https://de.wikipedia.org/wiki/Umgekehrter_Generationenvertrag] [all 24.04.2019]. Furthermore, students design initiatives that enable them to get involved in education, prevention, health care provision, health management and development aid as a contribution to regional and international health care delivery, education and further development [https://www.uni-wh.de/studium/studentische-initiativen/] [24.04.2019]. 

#### 2.4. Privacy and small size

With the goal of having the greatest possible freedom in university design, UW/H embarked on a path of non-profit privacy. This enables the University to implement new ideas regarding content and methodology comparatively quickly and easily, without being shackled to the state. However, this path also means that funding for expensive medical education has to be secured year after year, thereby providing a limited economic framework: “Out of money we have always been.” (Konrad Schily). 

The scarcity of funds in 1995 required students to assume responsibility for the entire organization in the form of a financial contribution. This led to the foundation of the purely student-run StudierendenGesellschaft, its aim being to provide tuition independently through a reverse generational contract, regardless of varying family incomes.

The small size of UW/H is a necessity to ensure affordability, yet simultaneously desirable in order to facilitate personal discussion between learners, teachers and employees. 

## 3. Results

The Witten Model Study Courses 1983-2018

### General

Student numbers: From 1983 and during its formative years, the University of Witten/Herdecke (UW/H) enrolled 24-27 medical students yearly. This number later increased to 42 students per year. As of 2008, the number of students has doubled per semester. Since the summer semester of 2019, enrollments have reached 84 students per semester, resulting in 164 students per year.

Financing: The UW/H is funded by various sources. More than a quarter of the revenue is generated via the financial contribution of the students. Other significant sources of income for UW/H are; donations and subsidies (17%), revenues from the dental clinic (approx. 15%), research funding (approx. 9%) and grants from the state of North Rhine-Westphalia. Since 2016, UW/H has also received funding from the University Pact III. The continuity of donations, sponsorship and third-party funding is of particular importance in terms of the long-term development and sustainability of the University. 

#### 3.1. The first “Witten Model Study Course MSG 1983” – to recognize and respect the dignity of the sick person

Gerhard Kienle called for the following regarding the design and content of medical studies at UW/H in his lecture to the medical Deans Symposium in 1982: “Training the doctor of tomorrow requires (...) the University to have autonomy with regards to action, judgment and decision making to ensure the scientific study of medicine that prepares a doctor:

- to recognize and respect the dignity of humans;
- to successfully provide personal assistance; and
- to develop independent judgment.” [[6], p. 354]

Basic framework conditions for the acquisition of these skills:

- Experiencing active medical practice;
- Training of perception, cognition and judgment;
- Developing the personality of the students,

These are all anchored in the curriculum of medical studies at the UW/H. 

##### 3.1. 1 Experiencing active medical practice: Training in the context of real patient care

In order to be able to provide the necessary “individual assistance” [10] for patients after completing their studies, training at the UW/H aims to make the competences actually required in medical practice tangible and learnable. The authentic context of patient contact is implemented through the integrated Perceptual Internship, the continuous General Practice Internship and the comprehensive Clinical Elective Blocks (a total of 54 weeks). 

##### 3.1. 2. The training of perception, cognition and judgment

In addition to experiencing medical practices, the ability to critically reflect on one’s own thinking and cognition as the basis for medical power of judgment and scientific faculty is the second cornerstone of Witten Didactics. The cross-disciplinary “studium fundamentale” (general studies) runs for an entire day each week for the duration of each semester and offers a space for practice, reflective thinking and cognitive activity with respect to liberal arts, the sciences an philosophy.

##### 3.1.3. Personality and personal development

The importance of personal development is the third cornerstone of medical studies at UW/H, as both a personal training goal and in the interactive and exchange-oriented University organizational development. It is strongly anchored in the general studies unit, the artistic and reflexive courses, and through scientific debate of epistemological, philosophical and historical issues. 

Individual design ideas, personal engagement and students’ energies are all embedded into UW/H’s development at both an organizational and curriculum level. This co-design role combined with a patient-centered curriculum creates a healthy development of students’ abilities. Most importantly, students learn the values and attitudes necessary in order to be able to adequately meet occupational and individual challenges. 

#### 3.2. The second “Witten Model Study Course MSG 1992” – Establishing problem-oriented learning (POL) 

From 1985 onward, Robert Wiedersheim (see above) was the second Dean of the Faculty of Medicine. In addition to the necessary development work, Wiedersheim also initiated and facilitated a process that dealt with the development of new approaches to teaching and learning that overcame the distinction between pre-clinical and clinical teaching [[11], p. 6]. Gradually, new teaching and learning formats were established, such as the “Living Anatomy” courses that accompany anatomy and physiology classes from the first semester onwards. Problem-Oriented Learning (POL) was systematically introduced in Germany for the first time as early as 1992, linked to clinical examination courses and clinical consultations in the respective POL patient histories. 

#### 3.3. The third Witten Model Study Course and the first official MSG 2000 Model Study Course – Training to be a medical person capable of lifelong learning

The third model study course in the UW/H’s history, and the first “officially recognized” course, used the new legal possibilities in 2000 to establish interdisciplinary, longitudinal formats (communication, ethics, science and medical economics as interdisciplinary topics), then to implement equivalent exam formats to replace state examinations. In addition there was more emphasis placed on training in General Practice (GP) settings. The previous central elements of medical education were retained. 

##### 3.3.1. Cornerstone 1: Training in outpatient General Practice settings

The previously established practice of regular internships in GP surgeries was further developed into a “GP Adoption Program”. This ran for the first semester onwards for a total of 5 weeks. The students received section-related tasks which had to be documented in a portfolio. Alongside the POL cases, GP consultation hours relating to the subject matter were introduced once a week. 

##### 3.3.2. Cornerstone 2: Longitudinal range of courses

A continuous, longitudinal range of courses were developed in four integrated curricula; communication, science and research, ethics, and health care systems and economics. These were combined with existing complementary optional formats from the field of integrative medicine, such as accompanying studies in Traditional Chinese Medicine (TCM). In addition, a student-initiated homeopathic working group began to draw increased attention, whilst preparation for the United States Medical Licensing Examination (USMLE) was introduced as a formal offer. 

In 2004 the Integrated Complementary Studies in Anthroposophic Medicine/Integrierte Begleitstudium Anthroposophische Medizin (IBAM) was introduced. It holds a special role due to its comprehensive design as an optional offer, its research training, and its search for innovative training formats. It seeks to supplement scientific methodology in medicine with idealistic philosophy, a spiritual view of humanity [12], and implementation of training formats with humanist characteristics [13]. Furthermore its aim is to research and test the contribution of anthroposophic medicine to the development of medical training and medicine as a whole [14]. The content of the IBAM is closely linked to the content of the central curriculum and integrates longitudinally into regular medical studies [15]. 

##### 3.3.3. Cornerstone 3: University internal, equivalent exam formats

- As equivalents to the first section of the state medical exam, three written case-based (Modified Essay Question, MEQ) and two practical (Objective Structured Clinical Examination, OSCE) exam formats have been established.
- Of the remaining examinations, 48% of the §27 exams are in the format of MC exams and approximately 40% are structured oral practical exams (Objective Structured Long Examination Record (OSLER); Objective Structured Clinical Examination (OSCE); Mini Clinical Examination, (Mini-CEX)). The rest is accounted for by reports and free text exams.
- The Progress Test in Medicine (Charité) that was traditionally completed at the beginning of each semester, is now used as a formative exam and internal evaluation tool for all semesters.

##### 3.3.4. Evaluation of the MSG 2000

The Model Study Course/Modellstudiengang (MSG 2000) in Medicine was approved by the Ministry of Health, Social Affairs, Women and Family of the State of North Rhine-Westphalia. The requirement of this approval was to continuously evaluate the degree course. 

In 2009 the MSG 2000 in Medicine was externally evaluated by an international commission of experts in accordance with the requirements of the Ministry of Health of North Rhine Westphalia. The results of the evaluation extended the regulations of UW/H until 2018. 

The MSG 2000 is regularly evaluated by the Center for Higher Education development (CHE) ranking. The data collated by CHE was evaluated in a 2010 study [16]. Medical graduates (1996-2002) who had graduated at least two years previously were interviewed online by the CHE. The UW/H graduates were found to be older, spent more time abroad, and were involved more frequently in clinical training. Nine questions related to professional medical practice were included in the survey and evaluated comparatively. UW/H graduates rated their medical studies as better in terms of independent learning and work, psychosocial skills, practical medical skills, teamwork, problem-solving skills, and interdisciplinary thinking. However, they judged their medical knowledge and scientific competence as inferior in comparison. 

The University was evaluated by the Science Council in 2005/06, 2011 and 2018 [17], [18], [19], [20]. In 2014, the Science Council issued recommendations for the further development of medical studies in Germany by way of an inventory of human medical model study courses (WR 4017-14). In an overall assessment, Manfred Prenzel summarized as follows: 

“All in all, the model study courses make a significant contribution to the development of medical studies in Germany. The introduction of the ‘model character’ framing has triggered a continuous process of change. It has allowed – also with regard to the reform of standard degree courses – creativity and a desire to shape faculties differently. The ‘model character’ framing can therefore be regarded as a success with regards to the expectations placed on it.” [https://www.wissenschaftsrat.de/download/archiv/pm_2114.pdf]

The assessment evaluated nine model courses, the largest observation period taking place at the University of Witten/Herdecke [21]. 

The MSG 2000 was assessed positively by the Science Council as part of a University-wide assessment. Council President Professor Wolfgang Marquardt stated the MSG 2000 was exemplary and could play a pioneering role for the whole of Germany [22]. 

In 2013, the model study course was awarded the Hartmannbund Faculty Prize for the best medical training, a prize that is awarded based on a nationwide online student survey [23]. 

Classes are evaluated by the students online at the UW/H at the end of each semester. Both the lecturers and the students who participated in the evaluation can access the results. The Vice Dean for Teaching then receives the raw data for further internal faculty evaluations. Furthermore, the evaluation results are used for leadership discussions between the Dean and the professors, and in addition for the further development of the degree courses. 

The above mentioned procedures are supplemented by semester-wide feedback interviews with students, and evaluation of the logbooks from the clinical elective blocks. The results are reported back to the lecturers and examiners, and discussed in cross-site subject area conferences.

In internal evaluations in 2013, students at UW/H rated the contribution of the medical general studies unit as good in terms of personal development, and as satisfactory with regards to technical and professional topics.

#### 3.4. The fourth Witten Model Study Course 2018^plus^ – Developing health together

The renewed reform considerations for the Model Study Course/Modellstudiengang (MSG 2018^plus^) were guided by a process-oriented view of the basic concepts of the medical system [24]: A person’s health, illness, suffering and healing are not seen as static states, but rather as dimensions of dynamic processes that require an integrated view of biological, mental-spiritual and societal social processes. Thus, the promotion of health is equal to caring for the sick. 

The conceptualization of future-oriented medical studies should therefore anchor around an integrated health care and health promotion system, which is multi-professional, team-based, patient-centered, process-oriented and increasingly out-patient-based and change-driven [25], [26]. 

##### 3.4.1. Educational goals of MSG 2018^plus^ – Gradual assumption of responsibility in real-life contexts of medical care provision

The Model Study Course/Modellstudiengang (MSG 2018^plus^), supplemented its previous training goal of ‘’...a medical person capable of lifelong learning’’, with “...a medical person capable of lifelong learning and acting”. Therefore the curriculum should not be solely based on an experiential spiral of knowledge and learning, but rather in addition embrace a spiral of action – in the sense of capacity for creative work and the ability to shape things [26]. 

The MSG 2018^plus^ integrates comprehensive self-designed learning spaces (longitudinal care for a patient, mentoring, peer teaching, track offers, etc.) with an underlying theoretical and practical clinical ability to act as a doctor. Practically speaking, this ability to act above and beyond the original elements of the model study course is achieved by gradually increasing the assumption of responsibility in real-life care settings of out-patient and in-patient elective placements. 

##### 3.4.2. Strengthening the cross-curricular, longitudinal thematic priorities 

In comparison to the MSG 2000, the new MSG 2018^plus^ further expands topics which have been anchored in the degree course in an interdisciplinary and longitudinal way. The MSG 2018^plus^ of the University of Witten/Herdecke (UW/H) was approved by the state of North Rhine-Westphalia and started in the 2018/2019 winter semester. It lists six thematic priorities (TP). 

##### TP 1: Outpatient health care 

Outpatient health care was redesigned in the MSG 2018^plus^. The early contact with GP care in the first semesters of study, which had proven viable in MSG 2000, has been further extended to include out-patient facilities, so-called “outpatient learning venues” (pediatrics, orthopedic surgeries, out-patient rehab, health authority offices), and “longitudinal care for a patient”. Interested students also have the choice to deepen their experience of primary care in three four-week “outpatient health care” tracks (see below). 

##### TP 2: Professional personal development – Inner Work (IAP) 

In this longitudinal curriculum, all previous elements of personal development are integrated and expanded. The goal is the development of an all-round professional person who cultivates skills in communication, reflection, teamwork and self-development. Practice and reflection are important experiences and tools on the path to lifelong development and self-education. 

##### TP 3: Interprofessional Education (IPE) 

A longitudinal offer of interprofessional courses with students from other health care professions satisfies the goal of UW/H to achieve effective interdisciplinary cooperation of all professions involved in health care. Joint courses (POL tutorials, patient examination courses and clinical consultations) with students of physiotherapy, health and nursing, speech therapy, etc. are designed and implemented at both the pre-clinical and clinical phase. 

##### TP 4: Working Scientifically 

The area of “working scientifically” will be significantly expanded in MSG 2018^plus^. Two six-week periods will focus on this topic, while other longitudinal events intensify training of this subject matter. Interested students are again given the opportunity to deepen their insights by taking the research track (see below). 

##### TP 5: Health system and care structures 

An interprofessional and exploratory teaching offer will be developed and implemented. This will enable students to familiarize themselves with existing care structures, institutions and professionals in the health sector, to explore approaches to further develop existing care structures (best practice examples), and for the students to develop and present to each other their own models for improving health care. 

##### TP 6: “Tracks” to offer interest-based choice in the compulsory curriculum

Three to four-week tracks will offer students an opportunity to focus on topics of individual interest more extensively and systematically than the previous model. Currently, tracks are planned for the following five thematic priorities: outpatient medicine, clinical medicine, research, digitalization in health care, and integrative medicine. 

## 4. Discussion and outlook

The first reform degree course at UW/H in 1983, the Model Study Course 2000, and the Model Study Course/Modellstudiengang (MSG 2018^plus^), all had the common aim of pursuing innovative ways of training doctors, whilst simultaneously contributing to a medical care system that aims to align itself with the dignity and individual needs of each patient and the respective societal requirements. 

Thus the following points were agreed upon at UW/H as the cornerstones of medical training:

1. The experience of medical practice combined with a gradual assumption of responsibility in the context of real-life medical care;
2. The training of perception, cognition and discernment: including scientific experience and knowledge in science, culture and humanities;
3. The personal development of students enabling them to grasp and reflect on questions of individual and normative attitudes and values.

The question of a timely implementation of core conditions geared toward the dignity of others in a medical education system remains a key challenge for today’s healthcare system and medical education. This is currently being discussed in the context of the increasing economization and standardization of medicine [27], [28], [29]. In addition to imparting knowledge and skills, the cornerstones of a medical education system reside in an orientation towards fundamental human values and attitudes. These include granting and safeguarding individual freedom of knowledge formation and reasoning of actions, the search for truth, and the commitment to one’s own social responsibility [30], [31]. These institutional ideals have remained unchanged throughout this development. Again and again they provide a compass for the continuous development of education oriented towards the necessities of changing societal conditions and requirements for medicine [32]. 

Both autonomy and independence from external (including governmental) requirements that the University aspires to are limited as they fall into a wider framework, defined by external conditions. These conditions include State University Law, training regulations, ministerial requirements, conditions of the Science Council, and state examinations. The discussion of these external influences leads to a productive tension which creates an intensive search for compromises and, above all, to quality assurance and benchmarking. 

The constant challenge of private sector financing has led to a broad shareholder structure and, moreover, to students assuming financial and organizational responsibility. This has led to a high level of identification of the students with the institution, and a shared social responsibility for the future generations of students. This was honored and specifically highlighted by the Science Council in 2011 [[19], p.14] 

From the outset, focus on the dignity and individuality of the patient led to a broader curricular focus on patient-centered medicine and education. This grew into a pedagogy centered on the learner as an individual, who orients oneself towards self- determined and intrinsically motivated learning and development models [33]. However, the teaching of these so-called ‘’soft skills’’ and other general skills (which were originally taught under general studies), did not appear to be specific enough to prepare students for personal medical competence. This observation led to the establishment of an integrated longitudinal curriculum in the first official model course which included a strand entitled “Communication and Ethics”. In the development of this strand, the focus in the current MSG 2018^plus^ centers around “Inner Work and Personal Development” (see above.)

Many of these approaches are shaped and implemented with relative ease due to the small size of the faculty and the Department of Human Medicine, the still manageable number of students, a good relationship between teachers and learners, and the combined strength of the communication of those involved. This closeness and personal interaction has also been established at other universities, such as the University of Massachusetts. This has been achieved through the establishment of “learning communities” that enable longitudinal contact between students and faculty. It is therefore feasible at larger universities.

The specific conditions for successful application (less focus on school-leaving marks, stronger focus on social commitment, ability to reflect, motivation for study, and specialized didactics) means that the curriculum at the UW/H cannot be directly compared with other universities. In general, students at UW/H are older and have more professional and international experience. Conclusively, studying at UW/H appears to be an excellent preparation for the workplace, particularly with regards to independent learning and work, psychosocial competences, practical medical skills, teamwork, problem-solving abilities, interdisciplinary thinking, and job satisfaction [16]. 

In its quest for new ways to further develop medical education, the UW/H has evolved over the past 36 years into a University that is part of a network of medical faculties working together on experiential and science-based developments, and in the reorientation of medical education. The UW/H is involved in research and teaching with many regional, national and international partners [34]. The small size and the non-governmental sponsorship of the UW/H opens up space to test and implement various new teaching and curriculum concepts. These can function as “laboratory scenarios” for testing prototypes that benefit state educational institutions [35]. 

In this sense, the new Model Study Course/Modellstudiengang (MSG 2018^plus^), sees itself as a template for forward-looking medical training that implements the essential goals of the Masterplan for Medicine 2020. In cooperation with the other faculties of the country, the aim is to contribute to the improvement of medical education, and thus the quality of patient care [36].

## Notes

Katja Frost and Friedrich Edelhäuser share lead authorship of this publication. 

## Competing interests

The authors declare that they have no competing interests. 
